# Transgenic Peanut (*Arachis hypogaea* L.) Overexpressing *mtlD* Gene Showed Improved Photosynthetic, Physio-Biochemical, and Yield-Parameters under Soil-Moisture Deficit Stress in Lysimeter System

**DOI:** 10.3389/fpls.2017.01881

**Published:** 2017-11-03

**Authors:** Kirankumar G. Patel, Radhakrishnan Thankappan, Gyan P. Mishra, Viralkumar B. Mandaliya, Abhay Kumar, Jentibhai R. Dobaria

**Affiliations:** ^1^Directorate of Groundnut Research (ICAR), Junagadh, India; ^2^Department of Biological Sciences, P. D. Patel Institute of Applied Sciences, Changa, India; ^3^Gujarat National Law University, Gandhinagar, India

**Keywords:** drought stress, peanut, lysimeter system, physio-biochemical parameters, growth-related traits, wilting symptoms

## Abstract

Peanut, an important oilseed crop, frequently encounters drought stress (DS) during its life cycle. In this study, four previously developed *mtlD* transgenic (T) peanut lines were used for detailed characterization under DS, at the reproductive stage using lysimeter system under controlled greenhouse conditions. In dry-down experiments, T lines maintained better photosynthetic machinery, such as, photosynthesis rate, stomatal conductance, transpiration rate, and SPAD (Soil-Plant Analyses Development) values, and had lower oxidative damage, including lipid membrane peroxidation and hydrogen peroxide and superoxide radical accumulation than WT, when exposed to 24 days of DS. WT plants had a more negative water potential (WP; up to −3.22 MPa) than T lines did (−2.56 to −2.71 MPa) at day 24 of DS treatment. During recovery, T lines recovered easily whereas 67% of WT plants failed to recover. In T lines, the rate of photosynthesis strongly and positively correlated with the transpiration rate (*r* = 0.92), RWC (*r* = 0.90), WP (*r* = 0.86), and total chlorophyll content (*r* = 0.75), suggesting its strong correlation with water retention-related parameters. Furthermore, yield parameters such as, pod weight and harvest index of T lines were up to 2.19 and 1.38 times more than those of WT plants, respectively. Thus, the significantly better performance of *mtlD* T peanut lines than of WT plants under DS could be attributed to the accumulation of mannitol, which in turn helped in maintaining the osmoregulation and ROS scavenging activity of mannitol and ultimately conferred water-economizing capacity and higher yield in T lines than in WT plants.

## Introduction

Peanut or groundnut (*Arachis hypogaea* L.), a glycophytic plant, is particularly vulnerable to water deficit stress or drought since it is usually grown under rainfed conditions on sandy soils (Banjara et al., 2011; Bhauso et al., 2014a). Nearly 70% of peanut-growing areas are located in semi-arid regions of the world, where drought is fairly common, limiting its production and productivity (Sarkar et al., 2014; Nawade et al., 2016). Reduction in the peanut productivity is nearly unpredictable since productivity is highly dependent on the timing, duration, and intensity of drought (Bhatnagar-Mathur et al., 2008; Sarkar et al., 2016). Globally, drought stress (DS) alone can cause an annual peanut yield loss of around 6–7 million tons, making the situation alarming and attracting utmost attention of the scientific community working on this crop (Bhatnagar-Mathur et al., 2014; Sarkar et al., 2014).

The adverse effect of DS on plants begins with a decrease in the transpiration rate (TR) and stomatal conductance (SC) and consequently leaf water potential (WP), resulting in overall imbalance in water relationships and the photosynthetic rate (Pn) (Farooq et al., 2009; Krasensky and Jonak, 2012; Ashraf and Harris, 2013; Osakabe et al., 2014). These effects are followed by a reduction in the turgor pressure, transpiration, and relative water content (RWC) and often the formation of reactive oxygen species (ROS), resulting in an increase in malondialdehyde (MDA) content and ultimately poor yield (Farooq et al., 2009; Akcay et al., 2010; Patel et al., 2016).

Under DS conditions, plants also undergo various molecular changes, resulting in increased expression of stress-associated genes responsible for synthesizing various regulatory proteins, detoxifying enzymes, proline, and compatible solutes (Chaves et al., 2009; Osakabe et al., 2014). Compatible solutes such as, polyols, betaines, sugars, and amino acids can stabilize macromolecules and cellular structures, maintain the turgor pressure in cells through osmotic adjustments and re-establish the cellular redox balance by scavenging free radicals (Krasensky and Jonak, 2012; Patel et al., 2016).

Mannitol is the most common naturally occurring polyol, which has been reported in over 100 plant species of various families, including Apiaceae, Oleaceae, and Rubiaceae (Conde et al., 2011), but not in peanut (Bhauso et al., 2014a). Mannitol, an important photosynthetic product, is a non-cyclic sugar alcohol and is one of the main phloem-translocated carbohydrate having functions such as, osmoregulation, coenzyme regulation, and free radical scavenging. The *Escherichia coli*-derived mannitol-1-phosphate dehydrogenase (*mtlD*) gene products catalyze the reversible conversion of fructose-6-phosphate to mannitol-1-phosphate, which is converted into mannitol through a naturally occurring non-specific phosphatase (Tarczynski et al., 1992). Mannitol accumulation by transforming the *E. coli mtlD* gene, which codes mannitol-1-phosphate dehydrogenase (MTD; EC 1.1.1.17), is associated with different abiotic stress tolerance in various plant species, including peanut (Nguyen et al., 2013; Bhauso et al., 2014a,b; Hema et al., 2014; Patel et al., 2016). ROS damage cellular structures, cause cell death, affect chlorophyll content, and reduce fertility (Chaves and Oliveira, 2004; Krasensky and Jonak, 2012), resulting in reduction in plant growth and ultimately plant yield.

Mannitol improves DS tolerance through scavenging of hydroxyl radicals and stabilization of macromolecular structures (Shen et al., 1997; Patel et al., 2016). It uses carbon skeletons and energy sources by which it plays a vital role in abiotic stress tolerance (Conde et al., 2011; Hema et al., 2014). Additionally, mannitol has water-like hydroxyl groups that mimic the structure of water and sustain an artificial sphere of hydration around macromolecules and function as osmoprotectants, providing tolerance to DS and resulting in better yield (Stoop et al., 1996; Conde et al., 2011).

Previously, we reported the successful transfer of the *mtlD* gene in peanut (cv. GG−20) and also demonstrated PEG-induced DS tolerance in transgenic (T) events in the hydroponic system (Bhauso et al., 2014b). Drought during reproductive stages such as, pegging and pod formation results in a drastic reduction of peanut yield and quality (Kambiranda et al., 2011). Thus, these stages are essential for DS tolerance analysis using the lysimeter system (Vadez et al., 2007, 2013; Ratnakumar et al., 2009). However, most of the previous DS experiments have been performed at the initial growth phase of *mtlD* T peanut plants, either in Petri plates, hydroponics or in small pots with least focus on the yield parameter (Nguyen et al., 2013; Bhauso et al., 2014a,b; Hema et al., 2014). The hypothesis of the present study is that the *mtlD* transgene in T peanut imparts DS tolerance even at maturity stages and improves peanut yield through a cascade of growth-regulating machinery.

Under DS conditions, root growth is accelerated in search of maximum available soil moisture content (Spollen et al., 2000; Durand et al., 2016). Small pot-based studies are suitable to study the initial discrimination of DS tolerance, but they may not provide a clear understanding of the actual performance of the transgenic plant in a field-like situation. Thus, this study was undertaken with the objective to evaluate *mtlD* T peanut lines at reproductive stages in the lysimeter system for various physio-biochemical and yield-related parameters, including root parameters, under DS conditions in a controlled containment facility.

## Materials and methods

### Plant material and creation of progressive soil moisture deficit conditions

Four transgenic (T) events (MTD1, MTD2, MTD3, and MTD4) in the T_5_ generation along with a wild-type (WT) line (cv. GG−20) were used for evaluating progressive soil moisture deficit stress or DS response through dry-down experiments. The T lines were confirmed for the presence of a transgene using gene-specific PCR primers (Figure S1). The seeds were grown in lysimeters, which were made of cylindrical PVC tubes of 1-m length and 20-cm diameter, containing ~42 kg of a soil mixture having soil:sand:FYM in 1:2:0.1 ratio. The experiments were conducted at ICAR-Directorate of Groundnut Research, Junagadh, Gujarat (India) in a controlled glasshouse under 16-h light conditions at 35 ± 2°C for 120 days.

At 40 days after sowing (DAS), a single plant, each from all four T lines and the WT line, was grown per lysimeter for both well-watered (WW) and DS treatments in three replications. Furthermore, at 40 DAS, the soil of both WW and DS lysimeters were saturated with water till field capacity (Colman, 1947). The soil surfaces were then covered using 500 g of plastic beads per lysimeter to prevent evaporation and were weighed at 3-day intervals between 09:00 and 10:00 h. Throughout the experiment, the WW plants were maintained at nearly 80% field capacity by supplementing water, which was lost through transpiration, at 3-day intervals. DS conditions were created by withholding irrigation until severe wilting symptoms appeared. Leaf samples of both WT and T lines were taken at 0, 10, and 24 days of DS imposition for physio-biochemical analysis. At the end of the experiment, all DS plants were re-watered with 200 mL of water for plant recovery up to 3 days at an interval of 24 h.

### Estimation of mannitol-1-phosphate dehydrogenase activity

MTD activity was measured as described by Iwamoto et al. (2003) using fresh leaf tissues at day 24 of DS. The MTD activity was expressed as fructose-6-phosphate reduction μmol mg^−1^ protein min^−1^, and the analyses were done using a Specord 200 spectrophotometer (Analytikjena, Germany).

### Photosynthesis, stomatal conductance, transpiration, and chlorophyll estimation

Photosynthesis, SC and transpiration were measured as per Banjara et al. (2011) on the third nodal leaflet using a portable photosynthesis system (LI-COR 6400, Li-Cor, Inc., Lincoln, NE, USA). The mean of three measurements obtained for each sample was used for analysis. The total chlorophyll content (TCC) in leaflets was measured at the center of each leaflet of every primary leaf using a portable chlorophyll measurement meter (SPAD−502PLUS, Konica Minolta, Japan) according to Banjara et al. (2011).

### Measurement of relative water content and leaf water potential

For RWC, both fresh weight (FW) and turgid weight (TW) of eight fresh leaf discs (1-cm diameter) were measured. These discs were then dried in a hot air oven (80°C, 72 h) and weighed until a consistent dry weight (DW) was obtained. RWC was calculated as RWC = [(FW–DW)/(TW–DW)] × 100 (Barrs and Weatherley, 1962). WP was measured as per Matthews et al. (1991) using a psychrometer CR7 (Campbell Scientific Inc., USA), which was first stabilized for 30 min, and then the leaf discs were placed in chambers equipped with a thermocouple for 30 min and data were collected for further analysis.

### Estimation of proline content

Free proline content was estimated using the protocol of Bates et al. (1973). Approximately 500 mg of fresh leaves was extracted with 5 mL of 3% sulphosalicylic acid and the homogenate was centrifuged at 10,000 × g for 10 min at 4°C. To 1 mL of supernatant, 1 mL of acid ninhydrin reagent and 1 mL of glacial acetic acid were added, mixed and incubated for 1 h at 100°C. The reactions were terminated on an ice bath. To the mixture, 2 mL of toluene was added and mixed vigorously, and the chromophore containing toluene was aspirated, warmed to room temperature and absorbance was measured at 520 nm. Proline content was calculated from a standard curve using 0–100 μg mL^−1^ of L-proline (Amresco, USA) and represented as μg g^−1^ of fresh tissue.

### Estimation of malondialdehyde content

The level of lipid peroxidation was measured in terms of MDA content using the thiobarbituric acid (TBA) reaction method (Heath and Packer, 1968). Leaf tissue was homogenized in trichloroacetic acid (10 mL, 0.1%, w/v) and centrifuged (15 min, 15,000 × g), and 1 mL of supernatant was mixed with 4 mL of TBA reagent (0.5%, w/v TBA in 20% w/v TCA). The mixture was heated (95°C, 30 min) and then cooled on ice and centrifuged (10 min, 10,000 × g). The absorbance of the supernatant was measured at 532 nm and the MDA content was calculated using the extinction coefficient of 155 mM^−1^ cm^−1^ and expressed as μmol g^−1^ FW.

### Detection of cell death and *in situ* localization of hydrogen peroxide and superoxide radicals

*In situ* localization of hydrogen peroxide (H_2_O_2_) and superoxide radicals (${\text{O}}_{2}^{-}$) and cell death were studied by histochemical staining using 3,3′-diaminobenzidine (DAB; Fryer et al., 2002) and nitroblue tetrazolium (NBT; Ramel et al., 2009) dyes and lactophenol trypan blue (Pogany et al., 2009), respectively. For this, 1 mg mL^−1^ of DAB (pH 3.8) was prepared in distilled water and 1 mg mL^−1^ of NBT was prepared in 10 mM potassium phosphate buffer (pH 7.8) containing 10 mM sodium azide. Lactophenol trypan blue (0.5 mg mL^−1^) solution was prepared in a phenol:glycerol:lactic acid:distilled water (1:1:1:1; v/v) mixture and the solution was diluted using an equal volume of absolute ethanol. Leaflets after 24 d of DS imposition, from both WW and DS plants were immersed in DAB, NBT, and lactophenol trypan blue solutions and kept in dark for 12 days. For lactophenol trypan blue staining, the leaflets were boiled for 1 min in the lactophenol trypan blue solution, bleached twice in an acetic acid:glycerol:ethanol (1:1:3; v/v/v) solution (80°C, 5 min) and stored in a glycerol:ethanol (1:4; v/v) solution until photographed. On the bleached leaflets, H_2_O_2_ was visualized as brown spots due to DAB polymerisation, ${\text{O}}_{2}^{-}$ as dark blue spots due to NBT precipitation and dead cells as blue spots.

### Measurement of soil moisture content, growth, and yield parameters

Soil moisture content was measured for samples in each lysimeter at 0, 10, and 24 days after DS imposition using the gravimetric method [(FW–DW)/DW × 100] (Black, 1965). The soil samples from 30 to 45-cm depths were taken using a screw auger and FW was measured, then the samples were dried at 72°C for 2 days and DW was measured. Different growth parameters, including lateral growth as root length (cm) and shoot length (cm), were measured for both WT and T plants after harvesting (120 DAS). Thereafter, the plants were dried (70°C, 72 h) and used for measuring traits including root and shoot biomass (g), pod weight (PW; g), and harvest index (HI,%). The HI was determined using the following formula: HI = (Economical yield/Biological yield) × 100. Where, PW was used as the “economical yield” and total biomass, including roots, as the “biological yield.”

### Statistical analysis

To determine the significance of variation between WT and T lines, the data were subjected to analysis of variance (ANOVA), and significant differences between WT and T lines were analyzed using Tukey's multiple ranges at 5% (LSD_*P* ≤ 0.05_) probability level. Each assay was performed in triplicate and the correlation coefficient was determined using PAST software (Paleontological Statistics, version 1.89).

## Results and discussion

### Mannitol-1-phosphate dehydrogenase activity

The role of mannitol accumulation as a way to overcome various abiotic stresses including DS has been proved in many crop species (Nguyen et al., 2013; Hema et al., 2014). Therefore, the *mtlD* gene coding for MTD was used for studying the imposition of drought tolerance in T peanut lines. Among various lines studied, WT peanut line showed almost nil MTD activity, whereas T lines showed significantly high MTD expression under both WW and DS conditions (Table 1). This not only confirmed the presence but also the expression of the transgene in the *mtlD* T peanut lines. Furthermore, among four T lines, significantly higher MTD activity was recorded in MTD1, MTD2, and MTD4, while least activity was recorded in MTD3 at day 24 of DS treatment (Table 1). Similar trend was also recorded for mannitol activity when measured at day 24 of DS treatment (Table S1). Thus, variations in MTD activity and mannitol content in different T lines could be due to the positional difference in the site of transgene integration (Bhauso et al., 2014b; Patel et al., 2016). Hence, both MTD activity and mannitol content clearly indicated their role in imparting DS tolerance to T lines as also reported by Patel et al. (2016) for salinity tolerance.

**Table 1:** Comparison of mannitol-1-phosphate dehydrogenase activity of WT and transgenic lines at 24 days under well-watered and drought stress conditions.

**Plant ID**	**Mannitol-1-phosphate dehydrogenase activity (μmol min^−1^ mg^−1^ protein)**	
	**Well-watered**	**Drought stress**
MTD1	0.062 ± 0.005^a^	0.034 ± 0.003^a^
MTD2	0.053 ± 0.003^a^	0.028 ± 0.001^b^
MTD3	0.027 ± 0.003^b^	0.016 ± 0.001^c^
MTD4	0.067 ± 0.004^a^	0.036 ± 0.002^a^
WT	0.002 ± 0.001^c^	0.004 ± 0.002^d^
LSD _(*P* = 0.05)_	0.01	0.005

*The mean values ±SE (n = 3) were followed by similar lower case letters within a column are not significantly different (P ≤ 0.05)*.

### Photosynthetic responses to drought stress

Photosynthesis, the fundamental, and complex physiological process in all plants, is severely affected by abiotic stresses (Osakabe et al., 2014) that alter the ultra-structure of organelles, concentration of pigments, including enzymes involved, and stomatal regulation (Chaves et al., 2009; Ashraf and Harris, 2013). Various transgenic plants having the *mtlD* gene are tolerant to various abiotic stresses (Maheswari et al., 2010; Nguyen et al., 2013; Hema et al., 2014), including peanut (Bhauso et al., 2014a,b; Patel et al., 2016). However, detailed account of the effect of mannitol accumulation on photosynthetic performance under water-limiting stress in *mtlD* T peanut is not known.

**Figure 1:**
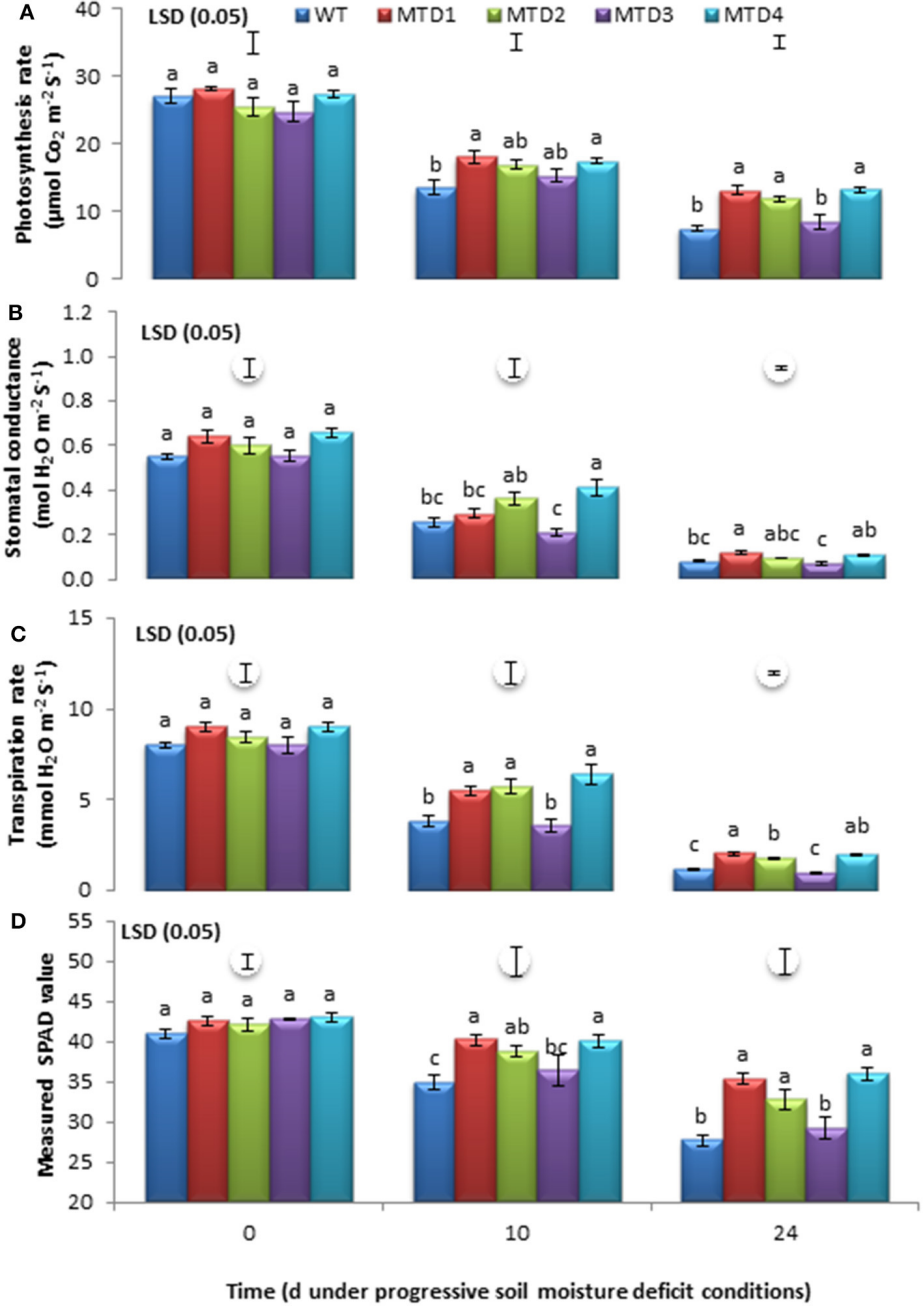
Estimation of photosynthetic rate **(A)**, stomatal conductance **(B)**, transpiration rate **(C)**, and SPAD values **(D)** of wild-type and transgenic lines at 0, 10, and 24 days under drought-stress conditions. The mean values ±SE (*n* = 3) followed by similar lower case letters within a column are not significantly different (*P* ≤ 0.05).

Data were recorded at days 10 and 24 of progressive DS development, and both WT and T lines showed a significant reduction in various photosynthetic parameters (Figure 1). However, no significant differences (*P* ≤ 0.05) were recorded under WW conditions (Figure S2). Furthermore, the rate of decline for these parameters was fairly steep in the WT line compared with the three T lines, MTD1, MTD2, and MTD4. The photosynthetic parameters were significantly higher in T lines than in the WT line (*P* ≤ 0.05), such as, Pn (30 and 71%; Figure 1A), SC (39 and 33%; Figure 1B), TRs (53 and 60%; Figure 1C), and SPAD values (14 and 25%; Figure 1D) measured at days 10 and 24, respectively. Similar results were obtained by Hu et al. (2005) and Sticklen et al. (2013) for higher SC and Pn in *mtlD* T poplar and maize plants, respectively, under salinity stress.

DS conditions inevitably generate ROS such as, H_2_O_2_, ${\text{O}}_{2}^{-}$, singlet oxygen ^1^O_2_, and hydroxyl radicals OH^−^, which have a deleterious effect on the activity of several photosynthesis-associated enzymes, including those for thylakoid electron transport, phosphorylation, and carboxylation (Osakabe et al., 2014; Cicero et al., 2015). Moreover, mannitol is well known for its OH^−^ scavenging activity, osmoregulation, and protection of thiol-regulated enzymes of the Calvin cycle (Shen et al., 1997; Chaves and Oliveira, 2004; Adrees et al., 2015). Thus, the improved performance of T lines compared with the WT line could be due to mannitol accumulation, which in turn has protected the photosynthetic machinery even under DS conditions.

### Relative water content and leaf water potential

RWC and WP are physiological indexes associated with water uptake by roots and water loss through transpiration. Hence, these parameters are considered as apt indicators when studying the water retention capacity of plants (Donovan et al., 2001; Bhauso et al., 2014a). As expected, no significant reduction (*P* ≤ 0.05) in RWC and water potential (WP) of WT and T lines was recorded under WW conditions (Table S2). However, a steady reduction in RWC and WP was recorded in both WT and T lines with the progression of DS duration (Table 2). Furthermore, the reduction was more prominent in WT line compared with T lines under DS conditions. In DS, three T lines, MTD1, MTD2, and MTD4, had a significantly higher RWC after 10 (76.6, 75.6, and 76.9%) and 24 days (59.3, 56.4, and 61.9%) compared with WT line (63.0 and 41.4%, respectively; Table 2). Similarly, in DS, WT line had a more negative WP of up to −3.22 MPa compared with T lines MTD1, MTD2, and MTD4, which had a negative WP ranging from −2.56 to −2.71 MPa at 24 days of DS (Table 2). Enhanced water retention parameters have been reported in *mtlD* T lines of wheat (Abebe et al., 2003), tomato (Khare et al., 2010), and maize (Nguyen et al., 2013) under various abiotic stresses.

**Table 2:** Comparison of relative water content, water potential, proline and malondialdehyde content in WT and transgenic lines at 0, 10 and 24 days under drought stress conditions.

**Plant ID**	**RWC (%)**			**Water potential (MPa)**			**Proline (μg g^−1^ FW)**			**MDA (μmol g^−1^ FW)**		
	**0 days**	**10 days**	**24 days**	**0 days**	**10 days**	**24 days**	**0 days**	**10 days**	**24 days**	**0 days**	**10 days**	**24 days**
WT	89.5 ± 3.0^a^	62.9 ± 3.0^c^	41.4 ± 4.6^d^	−0.96 ± 0.14^a^	−2.33 ± 0.09^a^	−3.21 ± 0.11^a^	201 ± 9.3^a^	1, 056 ± 71.6^b^	2, 385 ± 179^c^	2.88 ± 0.21^a^	4.97 ± 0.17^a^	7.13 ± 0.12^a^
MTD1	87.5 ± 3.2^a^	76.5 ± 4.2^a^	59.2 ± 2.5^ab^	−0.98 ± 0.12^a^	−1.82 ± 0.10^b^	−2.71 ± 0.13^b^	215 ± 10.4^a^	1, 321 ± 40.9^a^	3, 158 ± 50.9^b^	2.61 ± 0.24^a^	4.17 ± 0.16^bc^	5.72 ± 0.12^c^
MTD2	85.3 ± 3.8^a^	75.5 ± 2.9^a^	56.3 ± 2.6^b^	−1.03 ± 0.13^a^	−1.98 ± 0.15^ab^	−2.53 ± 0.06^b^	206 ± 14.5^a^	1, 173 ± 51.3^ab^	3, 876 ± 66.4^a^	2.52 ± 0.16^a^	3.82 ± 0.16^c^	5.97 ± 0.15^bc^
MTD3	87.4 ± 2.3^a^	68.9 ± 3.7^b^	46.5 ± 2.4^c^	−1.08 ± 0.11^a^	−2.10 ± 0.14^ab^	−3.24 ± 0.07^a^	200 ± 10.1^a^	1, 174 ± 51.3^ab^	2, 695 ± 46.5^c^	2.66 ± 0.17^a^	4.43 ± 0.24^ab^	6.45 ± 0.21^b^
MTD4	86.5 ± 5.3^a^	76.9 ± 2.6^a^	61.8 ± 2.3^a^	−0.95 ± 0.11^a^	−1.79 ± 0.12^b^	−2.55 ± 0.11^b^	198 ± 7.3^a^	1, 175 ± 51.3^a^	3, 841 ± 86.2^a^	2.86 ± 0.18^a^	3.78 ± 0.19^c^	5.54 ± 0.20^c^
LSD (*P* = 0.05)	5.79	5.21	4.72	0.384	0.387	0.322	33.30	146.54	310.92	0.58	0.57	0.48

*The mean ±SE (n = 3) followed by similar lower case letters within a column are significantly not different (P ≤ 0.05)*.

In cells, the water-like hydroxyl groups of mannitol mimic the structure of water (Stoop et al., 1996; Conde et al., 2011), and mannitol accumulation plays a vital role in the retention of WP (Mahajan and Tuteja, 2005; Khare et al., 2010) by which it prevents the intracellular loss of water and maintains the osmotic balance. Similarly, our results confirm that the better water retention capacity of T peanut lines over the WT line is because of mannitol accumulation, which in turn imparted tolerance to DS.

### Proline content

Proline is known to accumulate in plants under DS (Sarkar et al., 2014), which may play a role in neutralizing the negative effect of drought (Bhauso et al., 2014b). In DS, MTD1, MTD2, and MTD4 had more proline content (11–25% and 32–63% at days 10 and 24, respectively) compared with WT (Table 2). Similar type of enhanced proline accumulation was also reported in both *mtlD* and *DREB1A* T peanut plants (Bhauso et al., 2014b; Sarkar et al., 2014) under PEG-induced DS and in *AtDREB1A* and *MuNAC4* T peanut plants (Bhatnagar-Mathur et al., 2009; Pandurangaiah et al., 2014) in a dry-down experiment in pots. Thus, not only *mtlD* transgene but also other transgenes also induces proline formation when a T plant encounters various types of abiotic stresses. Because proline plays an important role in maintaining osmotic homeostasis and is a key component of cell wall proteins, its synthesis helps protect the plant from cellular dehydration (Pandurangaiah et al., 2014; Ravikumar et al., 2014).

### Malondialdehyde content and *in situ* localization of H_2_O_2_, ${\text{O}}_{2}^{-}$, and cell death detection

DS causes loss of cellular homeostasis accompanied by the excessive generation of ROS that results in lipid membrane peroxidation, which significantly increases the MDA content (Akcay et al., 2010). Compared with the WT line, there was a significantly lower MDA content and H_2_O_2_ and ${\text{O}}_{2}^{-}$ accumulation and cell death in T lines (Akcay et al., 2010; Patel et al., 2016), suggesting effective detoxification of H_2_O_2_ and ${\text{O}}_{2}^{-}$ and lower oxidative lipid injury in T plants (Table 2).

**Figure 2:**
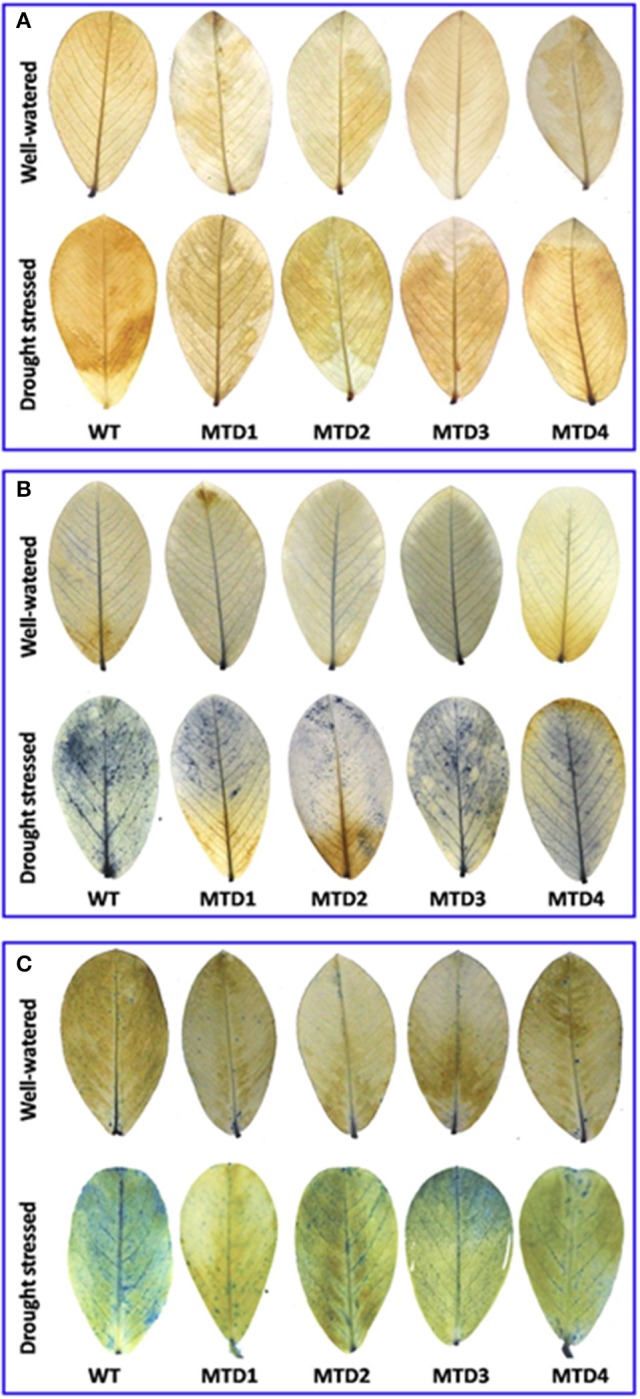
*In situ* localization of hydrogen peroxide **(A)** and the detection of superoxide radicals **(B)** and cell-death **(C)** in wild-type and transgenic lines under well-watered and drought stressed condition using DAB, NBT, and trypan blue staining, respectively.

Further, the extent of DS damage was analyzed as lipid membrane peroxidation, *in situ* accumulation of H_2_O_2_ and ${\text{O}}_{2}^{-}$ and cell death. Here, three T lines, MTD1, MTD2, and MTD4, had less MDA content (5.5–6.0 μmol g^−1^ FW) than the WT line (7.1 μmol g^−1^ FW), and were also found better in responding to DS. Histochemical *in situ* localization distinctly showed the lower accumulation of H_2_O_2_ (visualized as deep brown spots, Figure 2A) and ${\text{O}}_{2}^{-}$ (visualized as dark blue spots, Figure 2B) in T lines compared with the WT line under DS conditions. Similarly, cell death assay also evidently showed the significantly less number of dead cell patches (visualized as blue spots, Figure 2C) in T lines compared with the WT line.

### Soil moisture content and visual observations

**Figure 3:**
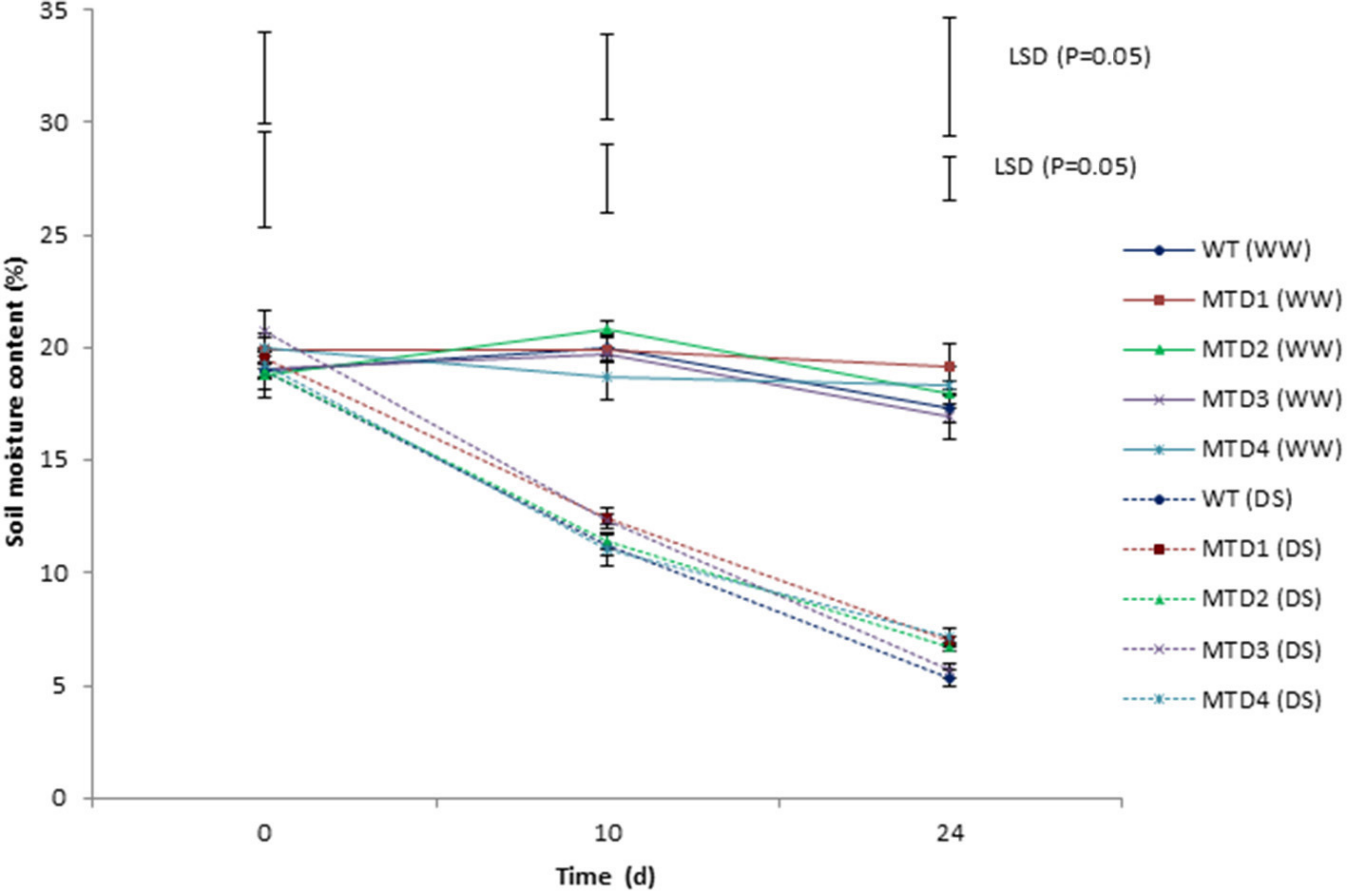
Comparison of soil-moisture content at 30 and 45 cm depths at 0, 10, and 24 days under well-watered and drought-stress conditions in wild-type and transgenic lines. Where, error bars on the line are SEM, while those on top represent LSD_(0.05)_.

**Figure 4:**
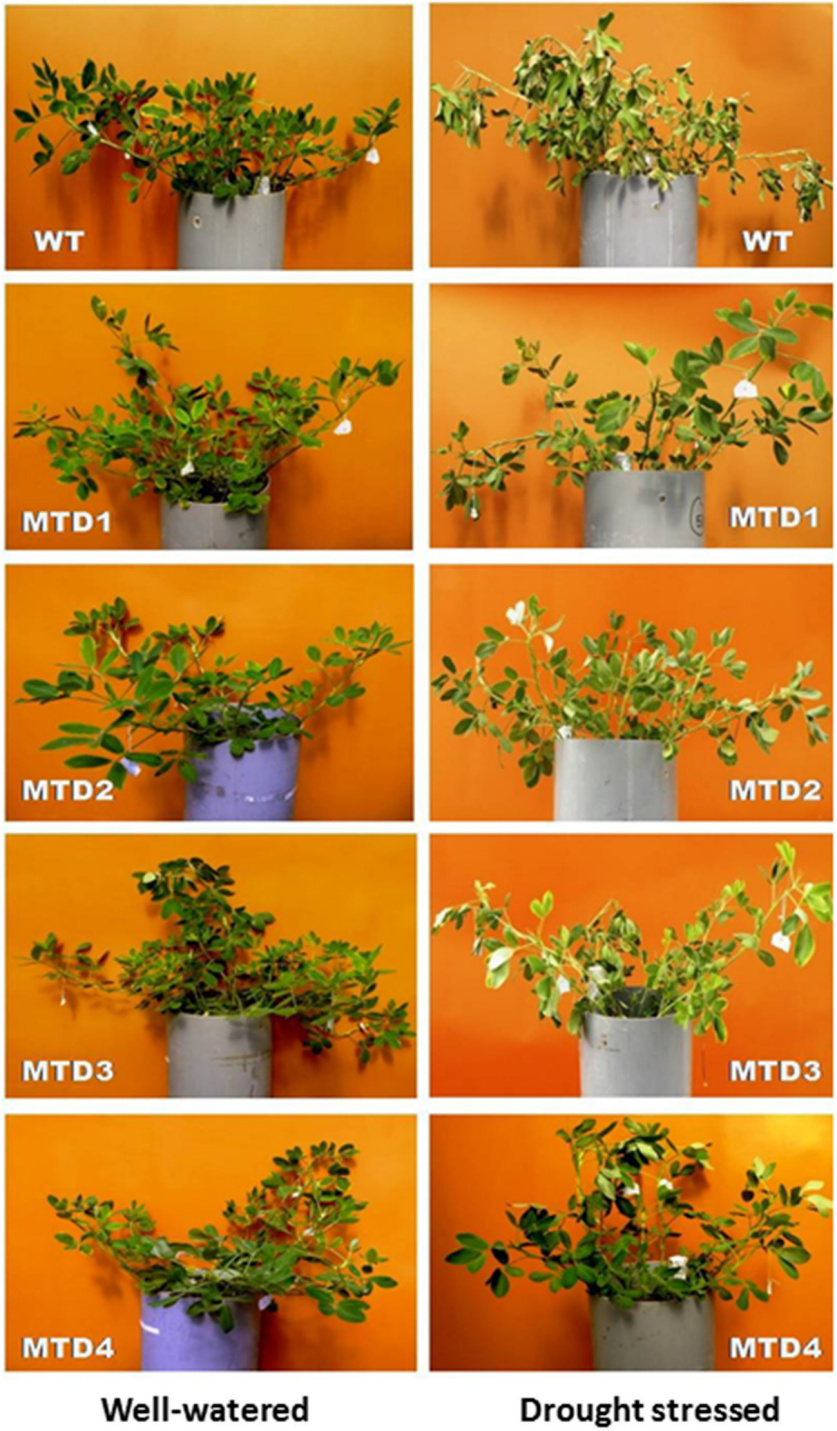
Comparisons of phenotypic traits between wild-type and transgenic lines at 24 days under well-watered and drought-stressed conditions.

DS was imposed by withholding irrigation for 24 days at reproductive stages such as, pegging and pod formation. The soil moisture content, which ranged from 18.9–20.8% initially, was reduced to 11.3–12.4 and 5.4–7.1% on days 10 and 24, respectively (Figure 3). All the T lines (except MTD3) showed improved phenotypic traits over WT. In WT, wilting symptoms started to appear on day 18, while in T lines, the symptoms appeared on day 20. Since on day 24 under DS condition, both T and WT plants started expressing severe wilting and dehydration symptoms; therefore, the plants were re-watered using 200 mL of water per day for 3 consecutive days for their recovery. Moreover, the severity of wilting was more pronounced in the WT line than in T lines (Figure 4). In addition, during recovery, the T lines recovered easily, while 67% of WT plants failed to recover, suggesting a more pronounced effect of DS on WT plants. Similar delayed wilting symptoms and fast recovery of T lines over WT after withdrawal of stress were reported by Bhatnagar-Mathur et al. (2014) and Sarkar et al. (2016) in *AtDRAB1A* T peanut plants. Bhauso et al. (2014a) also demonstrated lower water loss in T lines than in WT in an excised leaf assay.

The possible reasons for better survival and less wilting symptoms in T lines under DS conditions or even at same soil-moisture content could be the improved root architecture including a profuse and deep rooting system. This type of rooting pattern expectedly helped the T plants in better uptake of water from the deeper and wider soil volume. Similar results were also observed for *AtDREB1A* peanut line by Vadez et al. (2013) and Sarkar et al. (2016).

### Growth and yield parameters

Growth and yield parameters were also measured for both T and WT plants under DS and WW conditions after harvesting (120 DAS). T lines (except MTD3) under DS were relatively healthier than the WT line and showed notably enhanced growth parameters such root length (RL), root biomass (RB), shoot biomass (SB), pod weight (PW), and harvest index (HI) (Table 3). However, no significant differences were recorded for growth and yield parameters between WT and T lines under WW conditions (Table S3).

**Table 3:** Comparison of growth parameters of WT and transgenic lines after harvesting under drought stress conditions.

**Plant ID**	**SL**	**RL**	**SB**	**RB**	**PW**	**HI**
WT	51.3 ± 1.2^a^	61.4 ± 2.9^c^	23.6 ± 1.2^a^	2.9 ± 0.1^b^	5.4 ± 0.4^c^	21.8 ± 0.3^c^
MTD1	54.1 ± 1.3^a^	76.3 ± 2.0^ab^	24.4 ± 0.8^a^	3.3 ± 0.2^ab^	10.5 ± 0.6^ab^	29.1 ± 1.2^a^
MTD2	52.0 ± 1.3^a^	68.9 ± 3.0^bc^	23.8 ± 0.6^a^	3.0 ± 0.1^b^	8.3 ± 0.8^bc^	26.8 ± 1.3^ab^
MTD3	51.1 ± 1.3^a^	63.4 ± 3.5^c^	23.6 ± 0.4^a^	2.7 ± 0.3^b^	6.3 ± 0.7^c^	25.0 ± 2.0^bc^
MTD4	52.9 ± 1.0^a^	80.6 ± 2.2^a^	25.1 ± 0.5^a^	3.9 ± 0.3^a^	11.9 ± 1.0^a^	30.1 ± 1.9^a^
LSD (*P* = 0.05)	3.8	8.7	2.4	0.66	2.25	4.56

*The mean ±SE (n = 3) followed by similar lower case letters within a column are significantly not different (P ≤ 0.05). Where, SL, Shoot length; RL, Root length; SB, Shoot biomass; RB, Root dry Biomass; PW, Pod weight; HI, Harvest index*.

**Figure 5:**
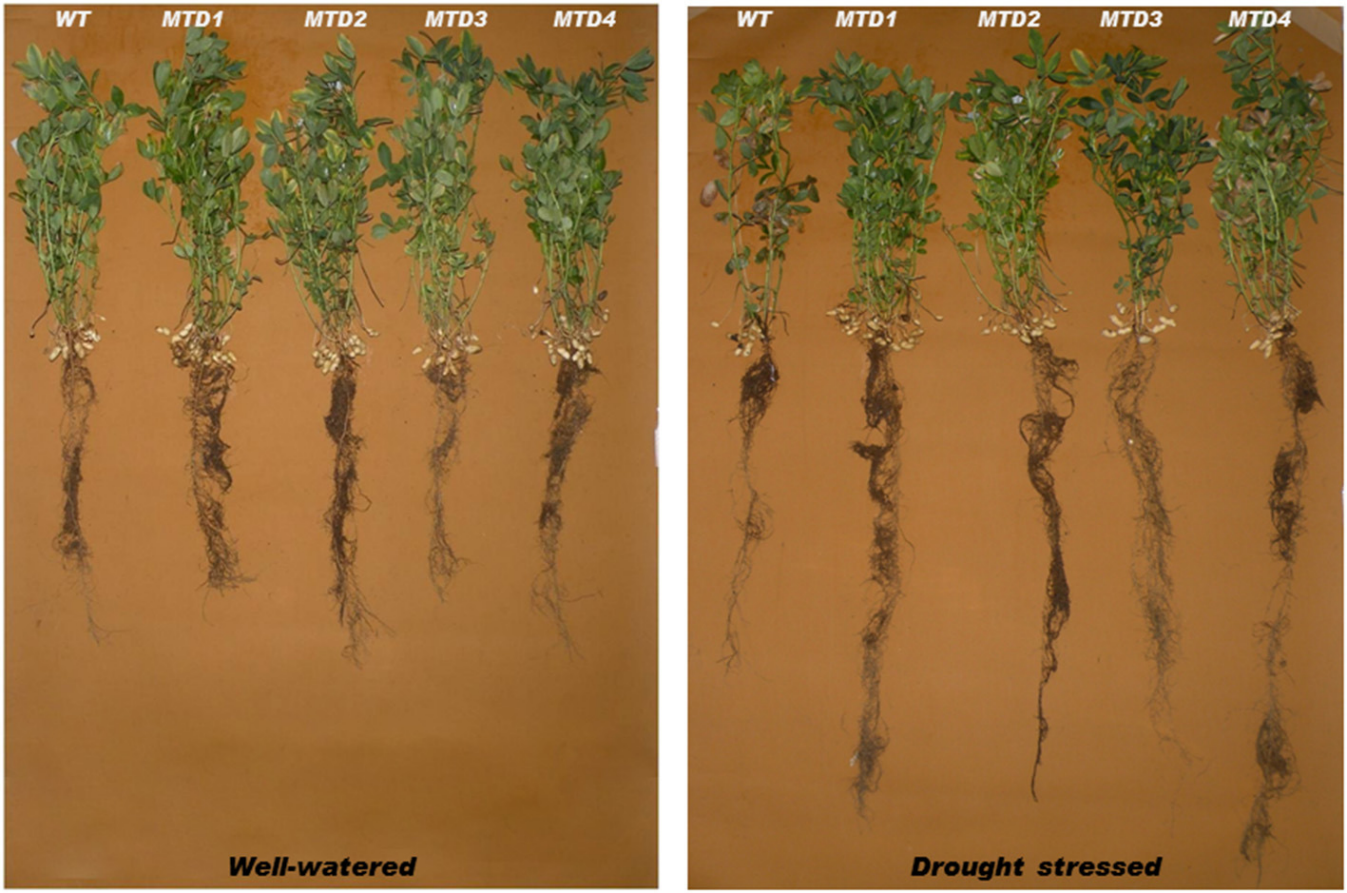
Variations in root growth between wild-type and transgenic lines under well-watered and drought-stressed condition after harvesting.

Although the characteristics of roots are generally evaluated using destructive sampling at set dates (Kashiwagi et al., 2006) in a lysimeter, they can be more easily and precisely estimated (Vadez et al., 2007; Bhatnagar-Mathur et al., 2008). Compared with WW conditions, the RL, and RB of WT peanut plants increased by 1.40-fold and 1.94-fold, respectively, under DS conditions. However, the increase in RL and RB for T peanut plants was 1.51–1.74-fold under WW conditions and 2.25–2.27-fold under DS conditions, respectively (Table 3; Figure 5). These results reiterate the fact that root growth was accelerated in search of maximum available soil moisture under DS conditions (Spollen et al., 2000; Durand et al., 2016). The SB of WT plants was reduced by 30%, whereas that of MTD1, MTD2, and MTD4 only by 6–18% (Table 3). Yield parameters such as, PW and HI were significantly higher for T lines than for the WT line under DS conditions (except MTD3). The PW and HI of T lines were 1.52–2.19-fold and 1.23–1.38-fold, respectively, more those of the WT line (Table 3). Overall, the growth parameter data in the present study were consistent with data in other *mtlD* expression studies in maize, sorghum, and wheat (Abebe et al., 2003; Maheswari et al., 2010; Nguyen et al., 2013). The improved growth and yield parameters of T plants than of WT plants could be attributed to the multiple effects of a large photosynthetic surface area, a better Pn, improved osmoregulation and a higher chlorophyll content due to mannitol accumulation (Banjara et al., 2011; Nguyen et al., 2013).

### Correlation analysis of various physio-biochemical and growth parameters

Linear correlation analysis was performed to find the relationship between different physio-biochemical parameters and growth-related traits under DS conditions at day 24 (Table 4). The Pn strongly and positively correlated with the TR (*r* = 0.92), RWC (*r* = 0.90), WP (*r* = 0.86), and TCC (*r* = 0.75), while MDA content negatively correlated with the Pn (*r* = −0.79). This suggests that the Pn is strongly correlated with water retention-related parameters and TCC. It also implies the role of mannitol accumulation in osmoregulation, which helps retain photosynthetic capacity under DS conditions (Shen et al., 1997; Conde et al., 2011; Bhauso et al., 2014b).

**Table 4:** Correlation analysis among various physio-biochemical and growth parameters.

	**Pn**	**SC**	**TR**	**TCC**	**PRO**	**MDA**	**RWC**	**WP**	**SL**	**RL**	**RB**	**SB**	**PW**	**HI**
Pn	1.00													
SC	0.73^*^	1.00												
TR	0.92^**^	0.75^*^	1.00											
TCC	0.75^*^	0.67^*^	0.62^*^	1.00										
PRO	0.68^*^	0.39	0.72^*^	0.54	1.00									
MDA	−0.79^**^	−0.64^*^	−0.64^*^	−0.89^**^	−0.60^*^	1.00								
RWC	0.90^**^	0.72^*^	0.85^**^	0.65^*^	0.71^**^	−0.91^**^	1.00							
WP	0.86^**^	0.61^*^	0.78^**^	0.52	0.83^**^	−0.83^**^	0.88^**^	1.00						
SL	0.67^*^	0.51^*^	0.55^*^	0.38	0.19	−0.46	0.40	0.35	1.00					
RL	0.61^*^	0.75^*^	0.80^*^	0.47	0.58^*^	−0.65^*^	0.83^**^	0.66^*^	0.58^*^	1.00				
RB	0.50^*^	0.62^*^	0.60^*^	0.47	0.49	−0.55	0.63^*^	0.53^*^	0.31	0.77^**^	1.00			
SB	0.77^**^	0.64^*^	0.85^**^	0.84^**^	0.72^*^	−0.81^**^	0.89^**^	0.61^*^	0.46	0.76^**^	0.70^*^	1.00		
PW	0.75^*^	0.82^**^	0.79^*^	0.86^**^	0.68^*^	−0.71	0.77^**^	0.40	0.34	0.84^**^	0.83^**^	0.59^*^	1.00	
HI	0.65^*^	0.56^*^	0.66	0.71^*^	0.20	−0.66	0.57^*^	0.56^*^	0.33	0.80^**^	0.76^**^	0.40	0.94^**^	1.00

Asterisks represent significant correlation at

* P ≤ 0.05 and

** *P ≤ 0.01*.

*For each parameter, average values of four T lines along with WT were used. Where, Pn, photosynthetic rate; SC, Stomatal conductance; TR, transpiration rate; TCC, total chlorophyll content; PRO, proline; MDA, malondialdehyde; RWC, relative water content; WP, water potential; SL, shoot length; RL, root length; SB, shoot biomass; RB, root biomass; PW, pod weight; HI, harvest index*.

In general, RWC and WP have strong to moderate positive correlation with RL, RB, TR, proline content, Pn, and SC. These results reiterate the fact that improvement in RWC and WP has a direct positive effect on the overall growth of T peanut plants (Sarkar et al., 2014). The significant positive correlation of proline content with WP (*r* = 0.83), TR (*r* = 0.72), and RWC (*r* = 0.71) confirmed that the free proline accumulation is responsible for better osmotic adjustments in T lines (Hayat et al., 2012).

Negative correlation of MDA was observed with RWC (*r* = −0.91), TCC (*r* = −0.89), WP (*r* = −0.83), SB (*r* = −0.81), and Pn (*r* = −0.79). This means that the cell membrane was severely damaged with increasing oxidative stress under DS conditions, which ultimately results in disturbed plant osmoregulation along with a reduced Pn, TCC, and SB. In the correlation studies of growth parameters, the RL showed a significant positive correlation (*r* = 0.76–0.84) with other growth-related parameters such as, RB, SB, PW, and HI. It means that improved root growth helps to absorb maximum available moisture from a wider soil volume and ultimately helps to improve other growth-related traits.

These findings support the hypothesis that mannitol accumulation not only improves the water retention capacity of T peanut plants but also protects the photosynthetic machinery during DS conditions (Shen et al., 1997; Chaves and Oliveira, 2004; Adrees et al., 2015). Thus, better DS tolerance of *mtlD* T peanut plants could be attributed to the osmoregulation and hydroxyl radical scavenging activities of mannitol, which could have imparted protection against oxidative injuries during DS conditions as also observed for *mtlD* T poplar (Hu et al., 2005) and maize (Sticklen et al., 2013).

### Poor performance of MTD3 event

As discussed in previous sections, for most of the physio-biochemical parameters, no significant difference was observed in the MTD3 line when compared with the WT line even at day 24 of DS. The plausible reasoning for the poor performance of MTD3 could be the lower expression of the transgene. Besides, several other factors, such as, the site of transgene integration, mutagenesis of transgene after insertion, pleiotropy, or transgene-induced endogenous silencing, may be present, which might have resulted in poor *mtlD* transgene expression (Yin and Malepszy, 2003; Kohli et al., 2010; Rahman et al., 2012). However, this finding needs further confirmation.

## Conclusions

Evaluation of four peanut *mtlD* T events along with a WT line was performed using a lysimeter system in a controlled containment facility, to simulate open-field conditions. The best performing T events, MTD1 and MTD4, will be used for open-field trials for their release as cultivars, especially for use in drought-prone areas. Furthermore, these identified T lines can also be used as a valuable pre-breeding resource to transfer the transgene using a backcross breeding approach into other favored genetic backgrounds, such as, backcross-derived Golden rice lines of Swarna (Nandakumar et al., 2008) and drought-tolerant *TaDREB3*-derived lines of wheat (Shavrukov et al., 2016). The results clearly signified that mannitol accumulation in the T lines played an important role in osmoregulation and hydroxyl radical scavenging, which facilitated maintenance of better photosynthetic machinery and lower oxidative damage. Thus, these DS-shielding mechanisms ultimately resulted in a better water-economizing capacity in *mtlD* peanut lines that grew well under DS conditions and gave a higher economic yield than the WT line.

## Author contributions

KP, Executed the experiments; RT, Conceived and helped in executing the experiments; GM, Prepared the manuscript; VM, Assisted in laboratory analysis; AK and JD, Helped in conducting the experiments.

### Conflict of interest statement

The authors declare that the research was conducted in the absence of any commercial or financial relationships that could be construed as a potential conflict of interest.

## Supplementary material

Figure S1 PCR based reconfirmation of transgenic lines using *mtlD* transgene specific primers in T5 generation. Where, lanes 1–4 are transgenic lines MTD 1, MTD 2, MTD 3, and MTD 4, respectively; P, Positive control (*mtlD* gene-construct); N, Negative control (WT: GG−20). The primer used was MtlD_694_ Fwd- 5′TCA ATC AGG TGG TAC TTG ATG C3′ and MtlD_694_ Rev−5′ TAG CGC TTG ATC AAT ACT GCA C3′ which gave 694 bp amplification only in the positive control and T lines.

Figure S2 Estimation of photosynthetic parameters of wild-type and transgenic lines under well-watered conditions. Where **(A)** Comparative photosynthetic rate, **(B)** Stomatal conductance, **(C)** Transpiration rate, and **(D)** SPAD values of WT and transgenic lines at 0, 10, and 24 days. The mean values ±SE (*n* = 3) followed by similar lower case letters within a column are not significantly different (*P* ≤ 0.05).

## Abbreviations

ROS: Reactive oxygen species
DS: Drought-stressed
WW: Well-watered
DAS: Days after sowing
MDA: Malondialdehyde
WT: Wild-type.
