# Supplementary figures and images for: Transgenic Peanut (Arachis hypogaea L.) Overexpressing mtlD Gene Showed Improved Photosynthetic, Physio-Biochemical, and Yield-Parameters under Soil-Moisture Deficit Stress in Lysimeter System
Source: Front Plant Sci. 2017 Nov 3;8:1881. doi: 10.3389/fpls.2017.01881 (PMC5675886; doi:10.3389/fpls.2017.01881)

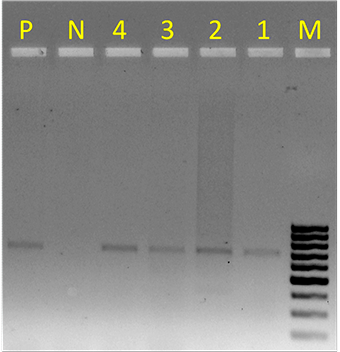

Supplement: Figure S1 — PCR based reconfirmation of transgenic lines using mtlD transgene specific primers in T5 generation. Where, lanes 1–4 are transgenic lines MTD 1, MTD 2, MTD 3, and MTD 4, respectively; P, Positive control (mtlD gene-construct); N, Negative control (WT: GG−20). The primer used was MtlD694 Fwd- 5′TCA ATC AGG TGG TAC TTG ATG C3′ and MtlD694 Rev−5′ TAG CGC TTG ATC AAT ACT GCA C3′ which gave 694 bp amplification only in the positive control and T lines. [file Image1.tif]

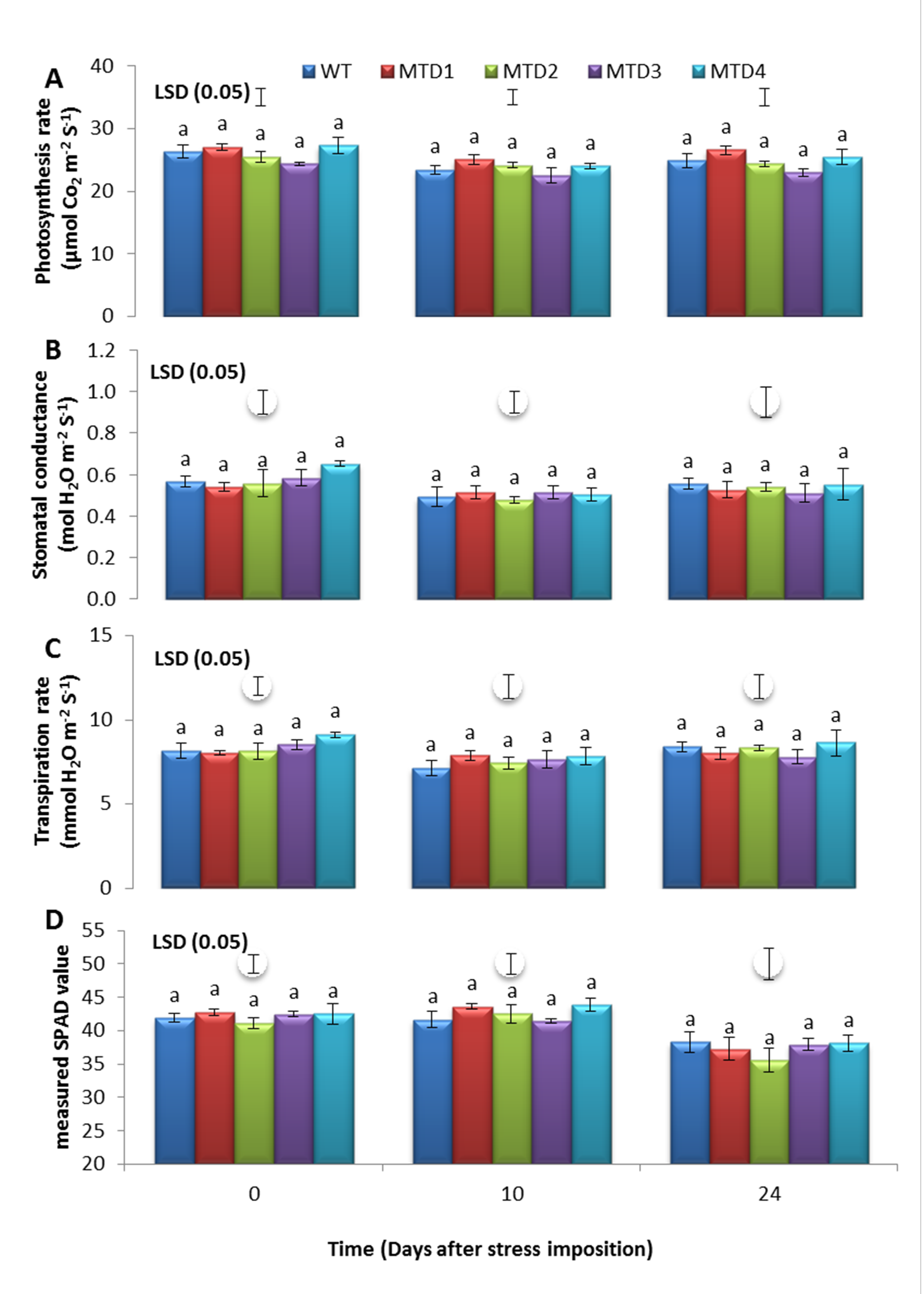

Supplement: Figure S2 — Estimation of photosynthetic parameters of wild-type and transgenic lines under well-watered conditions. Where (A) Comparative photosynthetic rate, (B) Stomatal conductance, (C) Transpiration rate, and (D) SPAD values of WT and transgenic lines at 0, 10, and 24 days. The mean values ±SE (n = 3) followed by similar lower case letters within a column are not significantly different (P ≤ 0.05). [file Image2.tif]
