# Supplementary material for: Transgenic Peanut (Arachis hypogaea L.) Overexpressing mtlD Gene Showed Improved Photosynthetic, Physio-Biochemical, and Yield-Parameters under Soil-Moisture Deficit Stress in Lysimeter System
Source: Front Plant Sci. 2017 Nov 3;8:1881. doi: 10.3389/fpls.2017.01881 (PMC5675886; doi:10.3389/fpls.2017.01881)
Supplement: Supplementary file 3 [file Table1.docx]

**Table S1. Comparison of mannitol accumulation in WT and transgenic lines at 24 days under well-watered and drought stressed conditions.**

| **Plant ID** | **Mannitol (µmol g^-1^ FW)** | |
| --- | --- | --- |
|  | WW (24 d) | DS (24 d) |
| MTD1 | 0.22 ± 0.014^a^ | 0.14 ± 0.014^a^ |
| MTD2 | 0.17 ± 0.007^b^ | 0.09 ± 0.009^b^ |
| MTD3 | 0.1 ± 0.012^c^ | 0.06 ± 0.007^b^ |
| MTD4 | 0.2 ± 0.007^ab^ | 0.15 ± 0.011^a^ |
| WT | ND | ND |
| LSD _(P=0.05)_ | 0.033 | 0.030 |

The mean±SE (n=3) followed by similar lower case letters as significantly not different (P≤0.05).
