# Supplementary material for: Transgenic Peanut (Arachis hypogaea L.) Overexpressing mtlD Gene Showed Improved Photosynthetic, Physio-Biochemical, and Yield-Parameters under Soil-Moisture Deficit Stress in Lysimeter System
Source: Front Plant Sci. 2017 Nov 3;8:1881. doi: 10.3389/fpls.2017.01881 (PMC5675886; doi:10.3389/fpls.2017.01881)
Supplement: Supplementary file 4 [file Table2.docx]

**Table S2. Comparison of relative water content, water potential, proline and malondialdehyde in WT and transgenic lines at 0, 10 and 24 d under well-watered conditions.**

| **Plant ID** | **RWC (%)** | | | **Water potential (MPa)** | | | **Proline (µg g^-1^ FW)** | | | **MDA (µmol g^-1^ FW)** | | |
| --- | --- | --- | --- | --- | --- | --- | --- | --- | --- | --- | --- | --- |
|  | 0 days | 10 days | 24 days | 0 days | 10 days | 24 days | 0 days | 10 days | 24 days | 0 days | 10 days | 24 days |
| WT | 88.4 ± 2.7^a^ | 86.1 ± 3.1^a^ | 81.8 ± 1.8^a^ | -9.93 ± 0.62^a^ | -12.46 ±1.29^a^ | -14.88 ±1.80^a^ | 195 ± 11.5^a^ | 295 ± 10.0^a^ | 310 ± 17.6^a^ | 2.49 ± 0.11^a^ | 2.89 ± 0.16^a^ | 3.05 ± 0.11^a^ |
| MTD1 | 86.9 ± 3.5^a^ | 85.6 ± 1.7^a^ | 83.2 ± 7.3^a^ | -10.37 ± 1.43^a^ | -12.78 ± 1.49^a^ | -15.38 ±0.97^a^ | 196 ± 8.8^a^ | 280 ± 5.8^a^ | 335 ± 16.2^a^ | 2.53 ± 0.16^a^ | 2.84 ± 0.19^a^ | 3.10 ± 0.17^a^ |
| MTD2 | 88.7 ± 4.1^a^ | 83.3 ± 2.5^a^ | 80.9 ± 3.9^a^ | -9.50 ± 1.18^a^ | -12.26 ± 1.00^a^ | -14.77 ±1.26^a^ | 216 ± 8.7^a^ | 293 ± 12.1^a^ | 313 ± 10.1^a^ | 2.75 ± 0.26^a^ | 2.49 ± 0.09^a^ | 3.20 ± 0.16^a^ |
| MTD3 | 90.1 ± 2.6^a^ | 84.2 ± 4.5^a^ | 81.3 ± 3.4^a^ | -9.87 ± 0.62^a^ | -11.85 ± 0.65^a^ | -15.38 ±1.10^a^ | 190 ± 13.2^a^ | 290 ± 12.6^a^ | 298 ± 6.0^a^ | 2.40 ± 0.19^a^ | 2.97 ± 0.15^a^ | 2.96 ± 0.17^ab^ |
| MTD4 | 88.5 ± 4.8^a^ | 83.3 ± 1.3^a^ | 84.3 ± 5.2^a^ | -10.49 ± 0.79^a^ | -11.85 ± 0.99^a^ | -14.54 ±1.09^a^ | 208 ± 6.0^a^ | 301 ± 9.3^a^ | 306 ± 11.7^a^ | 2.73 ± 0.18^a^ | 2.83 ± 0.20^a^ | 2.51 ± 0.12^b^ |
| LSD _(p=0.05)_ | 5.70 | 4.45 | 7.33 | 3.09 | 3.53 | 3.47 | 31.51 | 32.20 | 26.98 | 0.58 | 0.48 | 0.46 |

The mean ± SE (of three replicates; n=3) followed by similar lower case letter are not significantly different (P≤0.05).
