# Supplementary material for: Transgenic Peanut (Arachis hypogaea L.) Overexpressing mtlD Gene Showed Improved Photosynthetic, Physio-Biochemical, and Yield-Parameters under Soil-Moisture Deficit Stress in Lysimeter System
Source: Front Plant Sci. 2017 Nov 3;8:1881. doi: 10.3389/fpls.2017.01881 (PMC5675886; doi:10.3389/fpls.2017.01881)
Supplement: Supplementary file 5 [file Table3.docx]

**Table S3. Comparison of growth parameters of WT and transgenic lines after harvesting under well-watered condition.**

| **Plant ID** | **SL** | **RL** | **SD** | **RD** | **PW** | **HI** |
| --- | --- | --- | --- | --- | --- | --- |
| WT | 53.6 ± 1.4^a^ | 43.7 ± 3.5^a^ | 23.6 ± 1.2^a^ | 1.3 ± 0.1^a^ | 15.8 ± 0.8^a^ | 38.8 ± 1.6^a^ |
| MTD1 | 55.6 ± 1.9^a^ | 44.0 ± 1.9^a^ | 24.4 ± 1.7^a^ | 1.6 ± 0.1^a^ | 17.9 ± 0.3^a^ | 40.9± 2.0^a^ |
| MTD2 | 55.8 ± 3.4^a^ | 45.5 ± 2.2^a^ | 23.8 ± 1.5^a^ | 1.2 ± 0.2^a^ | 16.1 ± 1.1^a^ | 39.2± 2.0^a^ |
| MTD3 | 53.2 ± 1.7^a^ | 45.3 ± 1.4^a^ | 23.6 ± 1.4^a^ | 1.2 ± 0.1^a^ | 14.1 ± 0.6^a^ | 36.2± 0.3^a^ |
| MTD4 | 54.2 ± 2.7^a^ | 46.3 ± 3.8^a^ | 25.1 ± 1.6^a^ | 1.5 ± 0.1^a^ | 17.2 ± 1.7^a^ | 39.2± 1.1^a^ |
| LSD (p=0.05) | 7.4 | 8.7 | 4.7 | 0.45 | 3.21 | 4.84 |

The mean ± SE (of three replicates; n=3) followed by similar lower case letter are not significantly different (P≤0.05). Where, SL: Shoot length, RL: Root length, SB: Shoot biomass, RB: Root dry Biomass, PW: Pod weight and HI: Harvest index.
